# Proteomic landscaping of high‐grade serous ovarian carcinoma identifies stearoyl‐CoA desaturase 5 as a potential predictive biomarker for poly(ADP‐ribose) polymerase inhibitor response

**DOI:** 10.1002/ctm2.1693

**Published:** 2024-05-08

**Authors:** Se Ik Kim, Hyeyoon Kim, Kisoon Dan, Hong‐Beom Park, Cheol Lee, Hee Seung Kim, Hyun Hoon Chung, Jae‐Weon Kim, Noh Hyun Park, Dohyun Han, Maria Lee

**Affiliations:** ^1^ Department of Obstetrics and Gynecology Seoul National University College of Medicine Seoul Republic of Korea; ^2^ Department of Obstetrics and Gynecology Seoul National University Hospital Seoul Republic of Korea; ^3^ Proteomics Core Facility, Biomedical Research Institute Seoul National University Hospital Seoul Republic of Korea; ^4^ Biological Sciences Division Pacific Northwest National Laboratory Richland Washington USA; ^5^ Department of Biomedical Sciences Seoul National University Graduate School Seoul Republic of Korea; ^6^ Transdisciplinary Department of Medicine and Advanced Technology Seoul National University Hospital Seoul Republic of Korea; ^7^ Department of Pathology Seoul National University College of Medicine Seoul Republic of Korea; ^8^ Department of Pathology Seoul National University Hospital Seoul Republic of Korea; ^9^ Department of Medicine Seoul National University College of Medicine Seoul Republic of Korea

Dear Editor,

High‐grade serous ovarian carcinoma (HGSOC) has shown high recurrence and mortality rates despite treatment comprising cytoreductive surgery and chemotherapy.^1^ However, the recent introduction of poly(ADP‐ribose) polymerase inhibitors (PARPi) in the management of HGSOC significantly improved the prognosis.^2^ As more HGSOC patients receive PARPi treatment, accurately predicting the treatment response becomes crucial. In this study, we first establish the proteomic landscape of ovarian cancer according to PARPi response. Protein signatures were validated in the independent cohort, and preliminarily investigated their potential roles in PARPi resistance.

To identify protein signatures associated with PARPi resistance, we conducted an in‐depth quantitative proteomic analysis of formalin‐fixed paraffin‐embedded (FFPE) cancer tissues (*n* = 24) from platinum‐sensitive recurrent HGSOC patients using a tandem mass tag (TMT) 10‐plex (Supporting Information, Figure 1A). Patients’ clinicopathologic characteristics are presented in Table S1. All patients had germline and/or somatic *BRCA1/2* mutations and received either olaparib or niraparib for PARPi maintenance therapy. Based on the total duration of PARPi use, patients were divided into good response (≥12 months; *n* = 12) and poor response (< 12 months with disease progression; *n* = 12) groups. Patients in the good response group showed significantly better progression‐free survival (PFS) (Figure S1).

We used three strategies to identify prognostic protein candidates for treatment: statistical analysis, machine learning‐based feature selection, and weighted gene co‐expression network analysis (WGCNA) (Figure 1B–E). We identified a total of 8039 protein groups, and on average 6886 protein groups in each TMT experiment at a protein false discovery rate level of <1%, with 5906 proteins quantified across all samples (Table S2). The correlation coefficients of individual samples ranged from 0.961 to 0.999 (Figure S2). Between the good and poor response groups, no difference in tumor purity was observed. Principal component analysis revealed a small proteomic difference depending on response to PARPi, as well as other clinicopathological characteristics (Figure S3).

**FIGURE 1:**
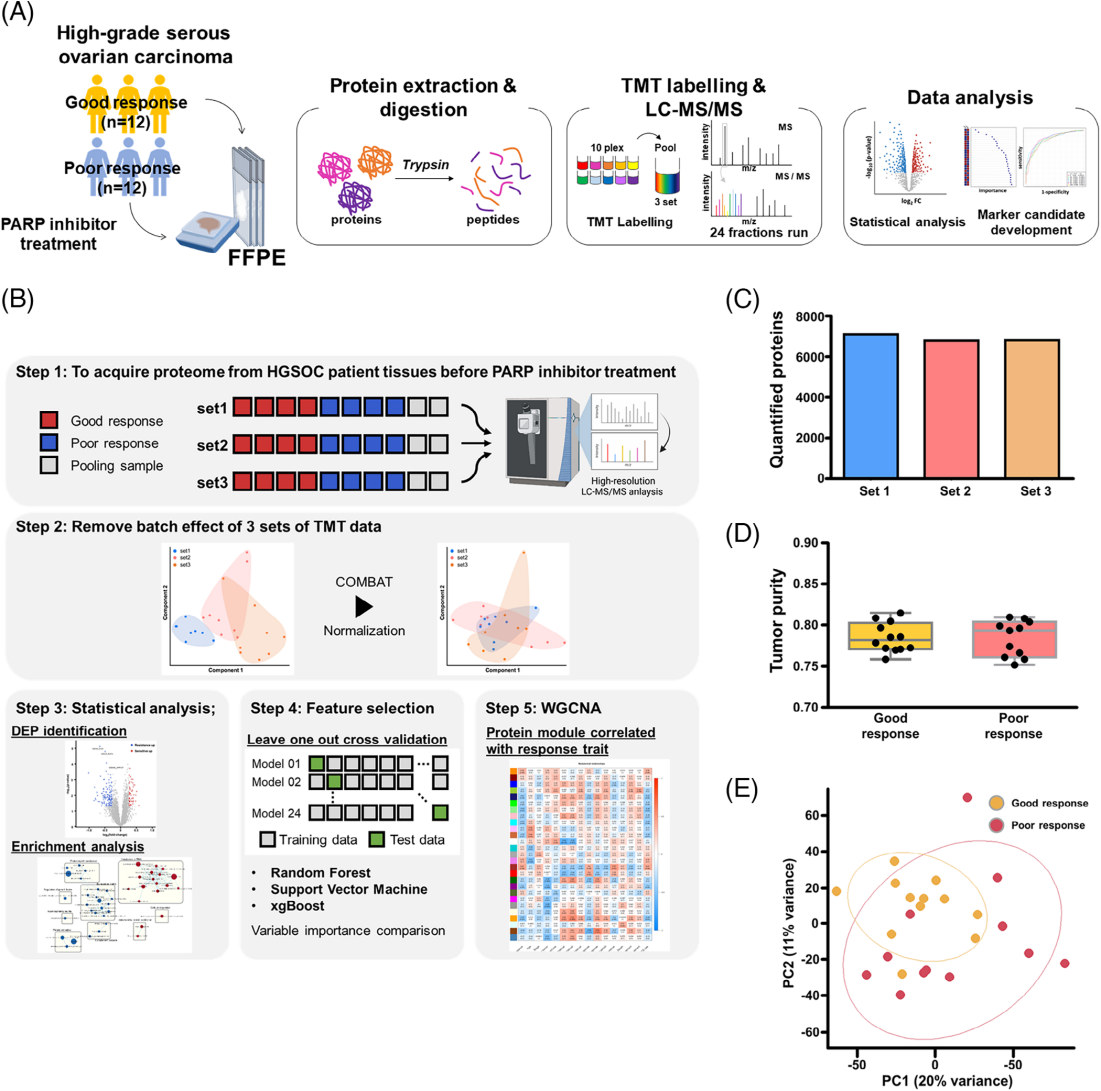
Study overview and formalin‐fixed paraffin‐embedded (FFPE) proteome characterization. (A) Summary of the experimental workflow and liquid chromatography‐tandem mass spectrometry (LC‐MS/MS) data acquisition for FFPE tissue proteomics. (B) Data analysis was conducted to unravel signatures depending on the responses to poly(ADP‐ribose) polymerase (PARP) inhibitors. (C) The number of quantified proteins in the three tandem mass tag (TMT) sets was determined using Proteome Discoverer (1% protein and peptide false discovery rate [FDR]). (D) Comparison of tumour composition between good and poor response group samples (*p *= .8117). (E) Principal component analysis (PCA) plot of log_2_ transformed normalized reporter ion intensities of 5906 proteins. Red, patients with poor response; orange, patients with good response.

According to PARPi treatment response, a total of 187 differentially expressed proteins (DEPs) were identified (*p *< 0.05, |fold‐change| > 1.2; Figure 2A and Table S3), with 54 and 133 exhibiting higher expression in the good and poor response groups, respectively (Figure S4 and Table S3). Enrichment analysis represented 36 biological processes and 20 Reactome pathways. In the good response group, mitochondrial dysfunction was significantly upregulated. Conversely, immune responses and extracellular matrix (ECM) organization were significantly upregulated in the poor response group. In Reactome, mitochondrial translational pathways and core pathways of mitochondrial protein synthesis were significantly upregulated in the good response group (Figure 2B). To gain more functional insight, we performed gene set enrichment analysis and found that ribosomal and infection‐related proteins were upregulated in the good response group, while ECM‐receptor proteins and coagulation‐related proteins were upregulated in the poor response group (Figure 2C and Figure S5).

**FIGURE 2:**
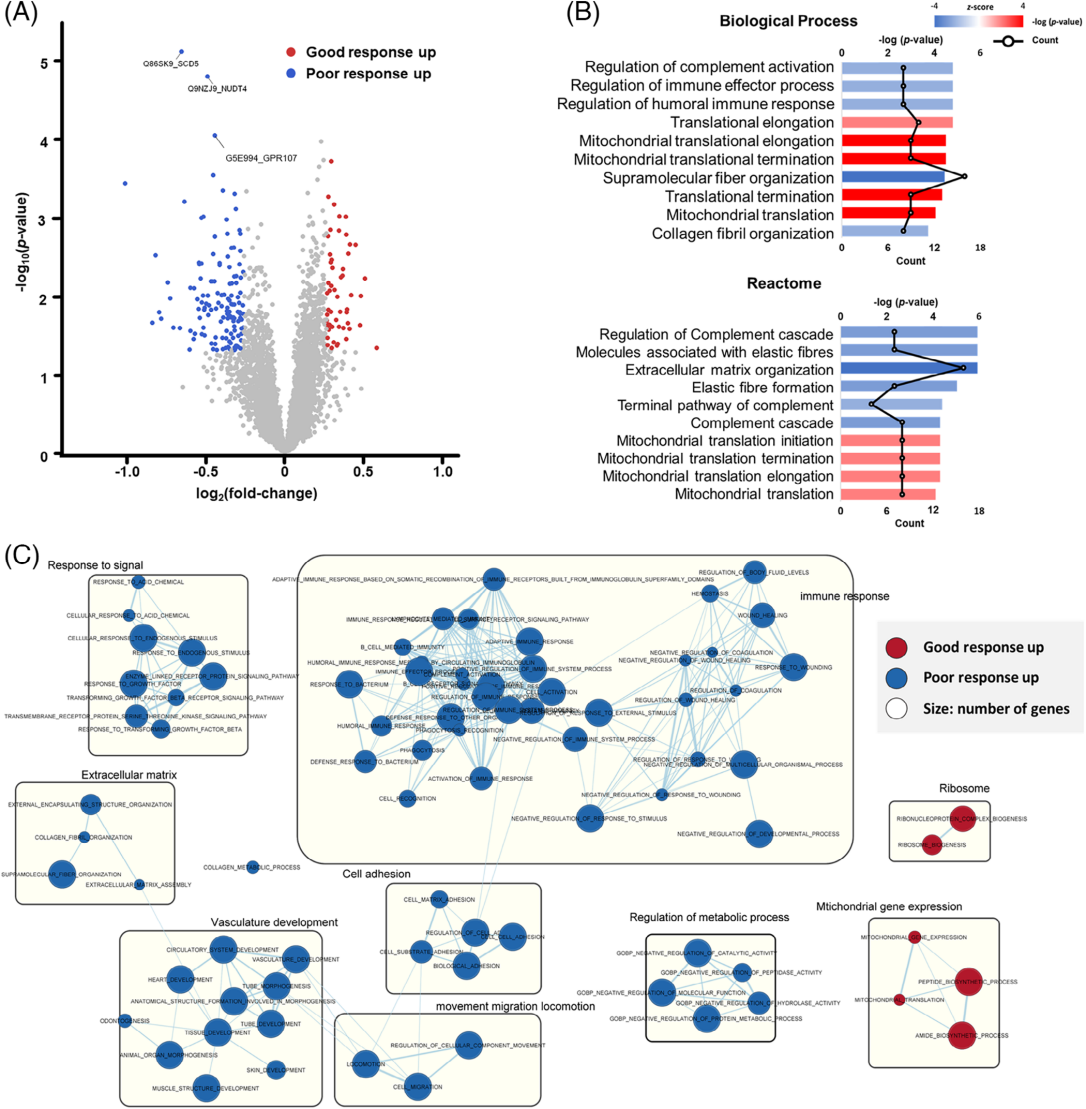
Identification of poly(ADP‐ribose) polymerase (PARP) inhibitor treatment response‐specific proteins. (A) Differentially expressed proteins between patients with good and poor responses, as determined by Student's t‐test (*p* < .05). Differences in good and poor responses are plotted against their ‐log_10_ (*p*‐value). Proteins significantly overexpressed in the good and poor response groups are indicated by the red and blue dots, respectively. (B) Enrichment analysis revealed that biological processes and pathways were significantly associated with proteins within the term. Terms represented by red bars (z‐score > 0) were predominantly associated with proteins that were significantly expressed in the good response group, whereas terms represented by blue bars (z‐score < 0) were primarily associated with proteins that were significantly expressed in the poor response group. (C) Gene‐set network proteomics features of sensitive and resistance groups. The enrichment map displays the terms enriched in the significantly expressed proteins. In the network, nodes represent terms, and the size of the nodes corresponds to the number of proteins enriched in those terms. Similar terms that share common proteins are connected and organized into clusters. The colour of the nodes reflects the differences in protein expression levels between the two groups. GSEA using C5 Gene Ontology (GO) gene sets focused on biological processes revealed pathways that exhibited significant alterations.

Next, we conducted feature selection with leave‐one‐out cross‐validation to identify protein signatures that stratify the response to PARPi. Random forest, SVM, and XGBoost yielded lists of top proteins selected by each respective algorithm (Figure 3A and Table S4). Among these, we identified three common proteins: stearoyl‐CoA desaturase 5 (SCD5), NUDT4 and GRP107 which were upregulated in the poor response group (Figure 3B and Figure S6).

**FIGURE 3:**
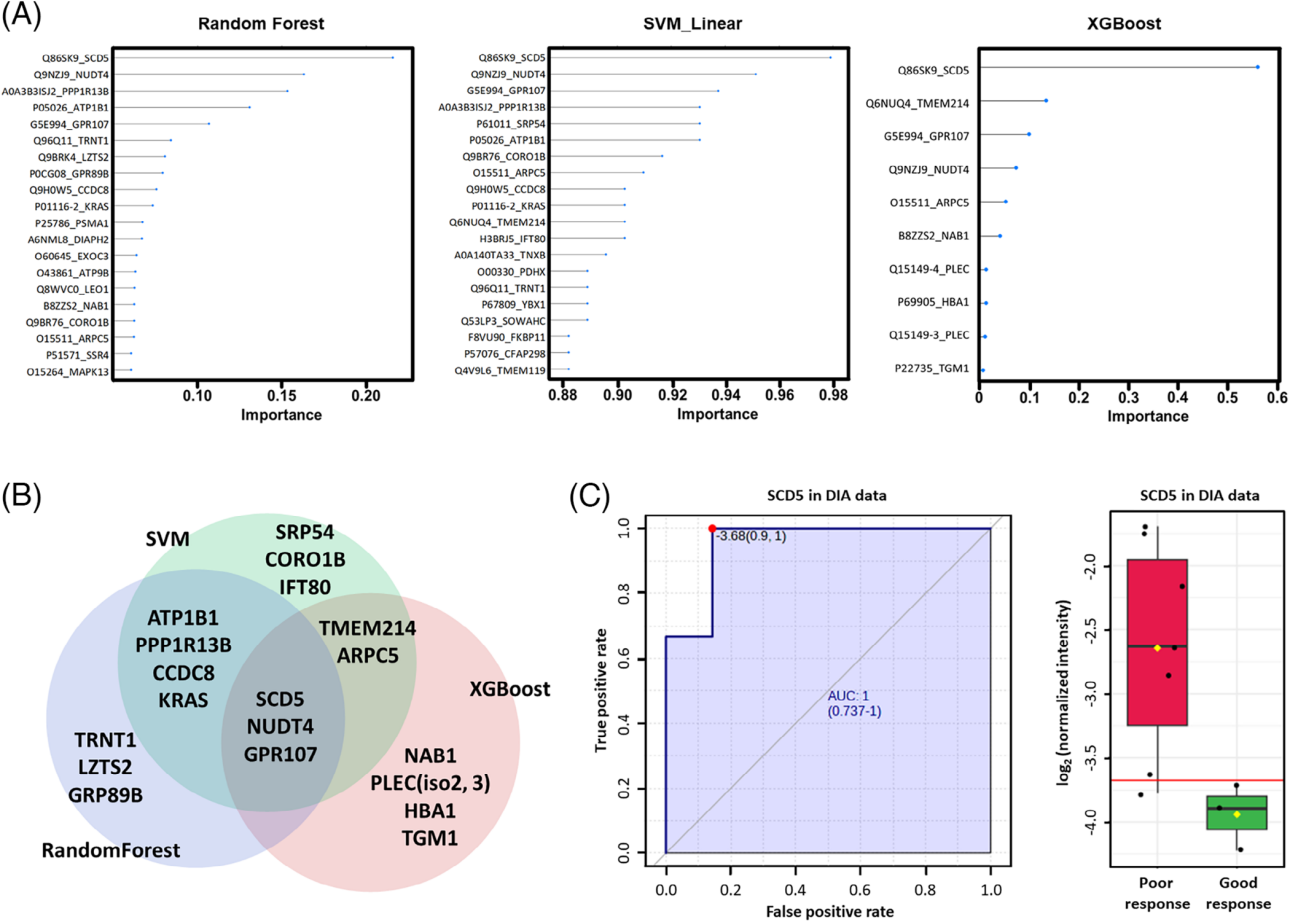
An outcome‐related signature composed of stearoyl‐CoA desaturase 5 (SCD5), NUDT4 and GPR107 was identified in platinum‐sensitive recurrent high‐grade serous ovarian carcinoma (HGSOC). (A) The top 20 proteins derived from the random forest and SVM linear, as well as the top 10 proteins from the XGBoost algorithm, are displayed in order of importance. (B) A Venn diagram displaying the 10 top‐ranked proteins from these three algorithms. (C) Receiver's operating characteristic (ROC) curve and boxplot of the data‐independent acquisition (DIA) data with expression levels of SCD5 (Poor response, *n* = 7; Good response, *n* = 3, *p *= .0352). High SDC5 expression levels are associated with an unfavorable response to PARPi in platinum‐sensitive recurrent HGSOC patients.

For validation, we performed data‐independent acquisition‐based proteomics using an independent set of FFPE tissues (three good and seven poor responders) (Table S5). All patients had germline and/or somatic *BRCA1* mutations and received PARPi maintenance therapy. TAPBP, NFIB, MAOB, APBA1, SLC25A19, TAP2 and SCD5 were significant DEPs in both discovery and validation experiments (Table S6). The SCD5 showed high predictive performance in identifying poor responders (area under the receiver operating characteristic curve, 0.952; Figure 3C).

To obtain an integrated view of protein expression and clinical parameters with discovery data, we subsequently conducted WGCNA (Figure S7). The correlation between the 27 modules and the clinical features was determined (Tables S7 and S8). The response to PARPi was significantly correlated with M5, M7, M9, M16 and M24 (Figure 4A). The M16 and platinum‐free interval (PFI) had a negative correlation, consistent with literature that links a short PFI to an unfavourable response to PARPi.^3^ Remarkably, the protein‐protein interaction map showed that the top‐ranked three proteins from the feature selection were closely associated with the M16 (Figure 4B). The Gene Ontology enrichment analysis (Bonferroni, *p *< 0.05) indicated that M16 was significantly linked to ECM organization and cell adhesion, whereas immune response was enriched in the M9, correlated with a good response to PARPi (Figure 4C and Figure S8).

**FIGURE 4:**
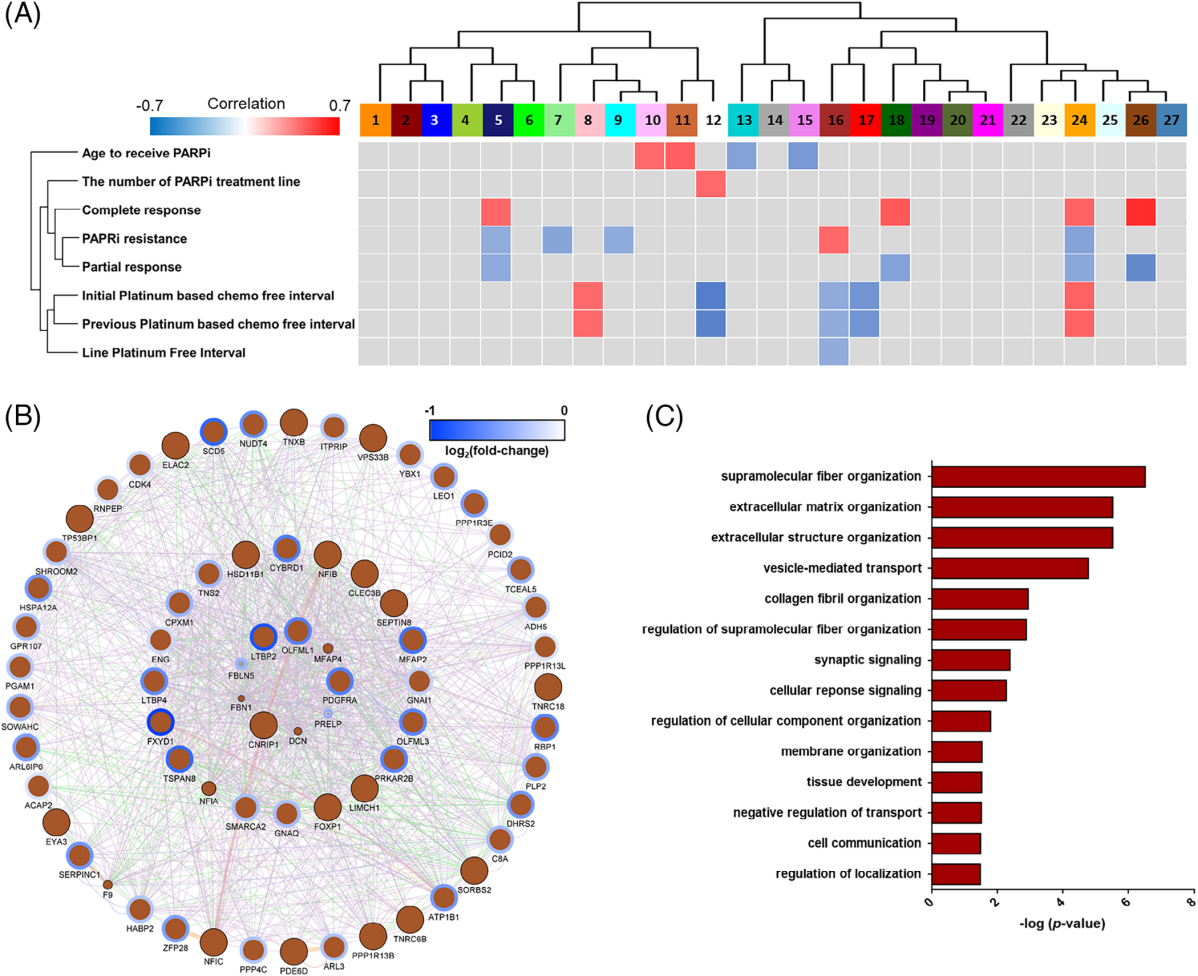
Network analysis of the formalin‐fixed paraffin‐embedded (FFPE) tissue high‐grade serous ovarian carcinoma (HGSOC) proteome. (A) WGCNA results for tandem mass tag (TMT) data. Biweight mid‐correlation analysis of colour modules with clinical traits of HGSOC. The strength of positive correlations is depicted in red and negative correlations in blue on a two‐colour heatmap. Only those with *p‐*values lower than .05 are displayed. (B) A protein–protein network of the M16 (brown) module. The colour of the contour line represents the fold change in proteome data, and the size of the circle corresponds to –log_10_ (*p‐*value). The nodes with black border lines are nonsignificant proteins. (C) Gene Ontology (GO) enrichment analysis was performed on the M16 (brown) module to gain insights into the biological significance of poly(ADP‐ribose) polymerase (PARP) inhibitor resistance.

Lastly, we preliminary explored the association between SCD5 and the PARPi resistance. SCD5 is involved in iron and lipid metabolism.^4^ Dysregulation of lipid synthesis and metabolism has been shown to regulate tumorigenesis and drug resistance in HGSOC.^5^ The SCD5‐related genes are involved in PPAR signalling pathways, which are regulated by PARPi in several cancers.^6^ Herein, we evaluated the potential role of SCD5 in olaparib resistance using OVKATE, an HGSOC cell line. Knockdown of SCD5 increased PARPi sensitivity in OVKATE cells. In HGSOC, PARPi resistance mechanisms involve drug efflux, alterations in PAR metabolism, mutational and epigenetic changes, protection of replication fork with stability, and restoration of the homologous recombination pathway.^7^, ^8^, ^9^ Notably, deficiency of SCD5 in OVKATE and OVCAR3 cells affected the expression of several genes associated with PARPi resistance, including DNA repair and the Switch/Sucrose non‐fermenting chromatin remodelling complex (Figure S9), increasing cell sensitivity to PARPi depending on double‐strand DNA repair mechanisms.

Moreover, the Kaplan‐Meier plot showed that high *SCD5* expression was significantly associated with worse PFS in HGSOC patients who received platinum‐based chemotherapy^10^ (Figure S10). Collectively, SCD5 overexpression was associated with resistance to PARPi and a poor prognosis in HGSOC patients.

In summary, we conducted the first systemic analysis of tissue proteome alterations in patients with HGSOC and proposed SCD5 as a predictive biomarker candidate for PARPi maintenance therapy. From a clinical viewpoint, for HGSOC patients who are identified as at high risk of showing poor response to PARPi maintenance therapy via pretreatment proteomics analyses, physicians may deter the use of PARPi, maximize the effect of PARPi combined with other therapy,^8^, ^9^ or recommend intensive surveillance during PARPi maintenance to detect recurrence earlier. Our findings broaden biological insights into predicting PARPi responses.

## AUTHOR CONTRIBUTIONS

**Se Ik Kim**: Methodology; Data curation; Formal Analysis; Writing—Original Draft and Writing—Review & Editing. **Hyeyoon Kim**: Methodology; Data Curation; Visualization; Formal Analysis; Writing—Original Draft and Writing—Review & Editing. **Kisoon Dan**: Methodology; Formal Analysis; Investigation and Writing—Review & Editing. **Hong‐Beom Park**: Formal Analysis and Validation. **Cheol Lee**: Methodology; Data Curation and Investigation. **Hee Seung Kim**: Investigation; Validation and Writing—Review & Editing. **Hyun Hoon Chung**: Investigation; Validation and Writing—Review & Editing. **Jae‐Weon Kim**: Investigation; Validation and Writing—Review & Editing. **Noh Hyun Park**: Investigation; Validation and Writing—Review & Editing. **Dohyun Han**: Conceptualization; Methodology; Formal Analysis; Resources; Software; Writing—Original Draft and Writing—Review & Editing. **Maria Lee**: Conceptualization; Methodology; Resources; Software; Formal Analysis; Funding Acquisition; Project Administration and Writing—Review & Editing.

## CONFLICT OF INTEREST STATEMENT

The authors declare no conflict of interest.

## ETHICS STATEMENT

This study was approved by the Institutional Review Board of Seoul National University Hospital (No. H‐2011‐127‐1173). We conducted the study in accordance with the principles of the Declaration of Helsinki and its amendments. All patients agreed to donate biospecimens for scientific purposes and provided written informed consent.

## Supporting information

Supporting information

Supporting information

Supporting information

Supporting information

Supporting information

Supporting information

Supporting information

Supporting information

Supporting information

Supporting information

Supporting information

Supporting information

## Data Availability

The data that support the findings of this study are available from the corresponding authors upon reasonable request. The mass spectrometry proteomics data were deposited in the ProteomeXchange Consortium via the PRIDE partner repository with the dataset identifier PXD044703.

## References

[1] Matulonis UA , Sood AK , Fallowfield L , Howitt BE , Sehouli J , Karlan BY . Ovarian cancer. Nat Rev Dis Primers. 2016;2:16061. 27558151 10.1038/nrdp.2016.61 PMC7290868

[2] Ledermann JA . PARP inhibitors in ovarian cancer. Ann Oncol. 2016;27(1):i40‐i44. 27141070 10.1093/annonc/mdw094

[3] Fong PC , Yap TA , Boss DS , et al. Poly(ADP)‐ribose polymerase inhibition: frequent durable responses in BRCA carrier ovarian cancer correlating with platinum‐free interval. J Clin Oncol. 2010;28(15):2512‐2519. 20406929 10.1200/JCO.2009.26.9589

[4] Konstorum A , Lynch ML , Torti SV , Torti FM , Laubenbacher RC . A systems biology approach to understanding the pathophysiology of high‐grade serous ovarian cancer: focus on iron and fatty acid metabolism. Omics. 2018;22(7):502‐513. 30004845 10.1089/omi.2018.0060 PMC6059353

[5] Zhao G , Tan Y , Cardenas H , et al. Ovarian cancer cell fate regulation by the dynamics between saturated and unsaturated fatty acids. Proc Natl Acad Sci U S A. 2022;119(41):e2203480119. 36197994 10.1073/pnas.2203480119 PMC9564215

[6] Oatman N , Dasgupta N , Arora P , et al. Mechanisms of stearoyl CoA desaturase inhibitor sensitivity and acquired resistance in cancer. Sci Adv. 2021;7(7). 10.1126/sciadv.abd7459 PMC7875532 33568479

[7] Kim DS , Camacho CV , Kraus WL . Alternate therapeutic pathways for PARP inhibitors and potential mechanisms of resistance. Exp Mol Med. 2021;53(1):42‐51. 33487630 10.1038/s12276-021-00557-3 PMC8080675

[8] Dias MP , Moser SC , Ganesan S , Jonkers J . Understanding and overcoming resistance to PARP inhibitors in cancer therapy. Nat Rev Clin Oncol. 2021;18(12):773‐791. 34285417 10.1038/s41571-021-00532-x

[9] Miller RE , El‐Shakankery KH , Lee J‐Y . PARP inhibitors in ovarian cancer: overcoming resistance with combination strategies. J Gynecol Oncol. 2022;33(3). 10.3802/jgo.2022.33.e44 PMC9024188 35320891

[10] Győrffy B . Discovery and ranking of the most robust prognostic biomarkers in serous ovarian cancer. Geroscience. 2023;45(3):1889‐1898. 36856946 10.1007/s11357-023-00742-4 PMC10400493

