# Supplementary figures and images for: Proteomic landscaping of high‐grade serous ovarian carcinoma identifies stearoyl‐CoA desaturase 5 as a potential predictive biomarker for poly(ADP‐ribose) polymerase inhibitor response
Source: Clin Transl Med. 2024 May 8;14(5):e1693. doi: 10.1002/ctm2.1693 (PMC11079157; doi:10.1002/ctm2.1693)

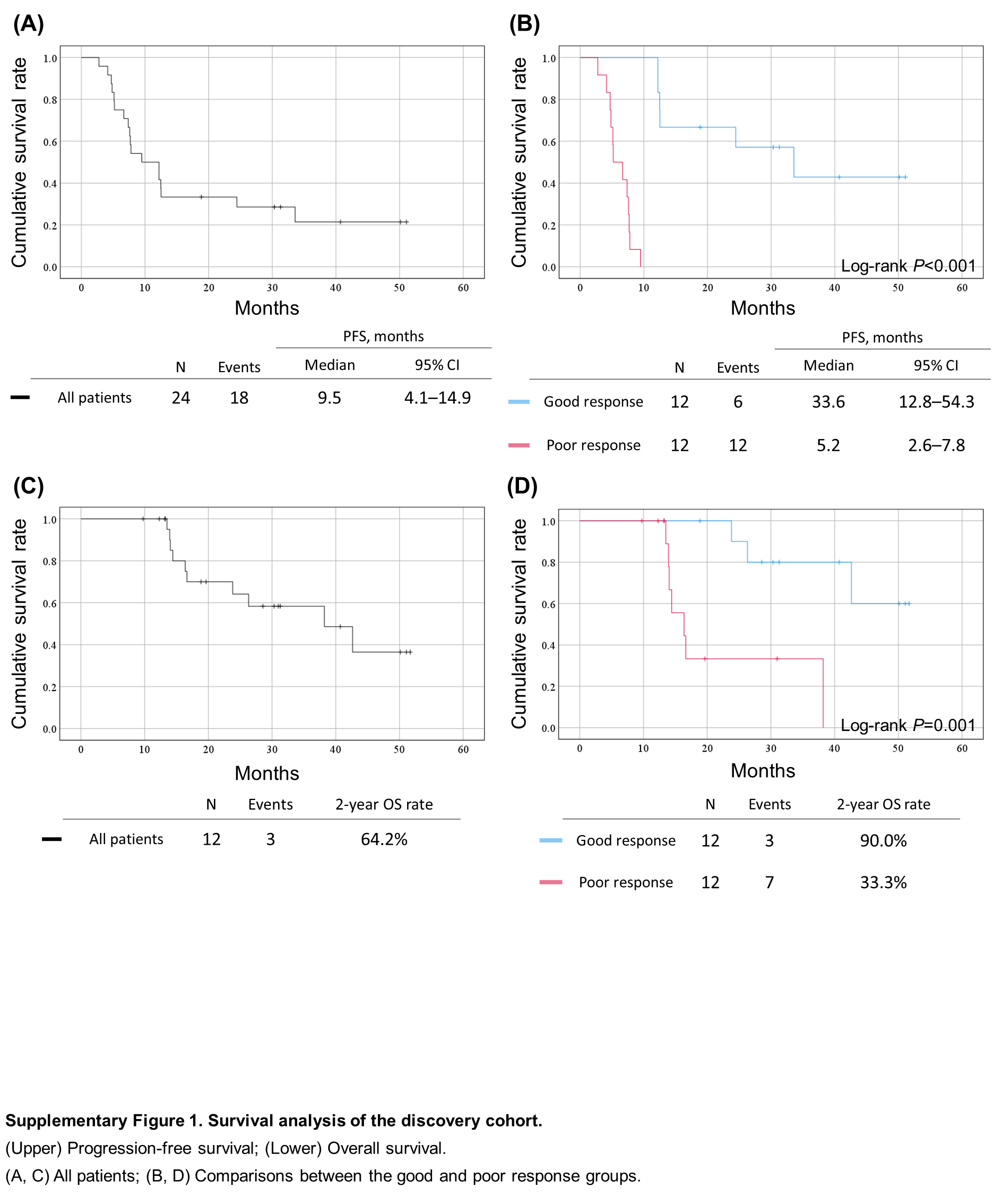

Supplement: Supplementary file 1 — Supporting information [file CTM2-14-e1693-s007.PNG]

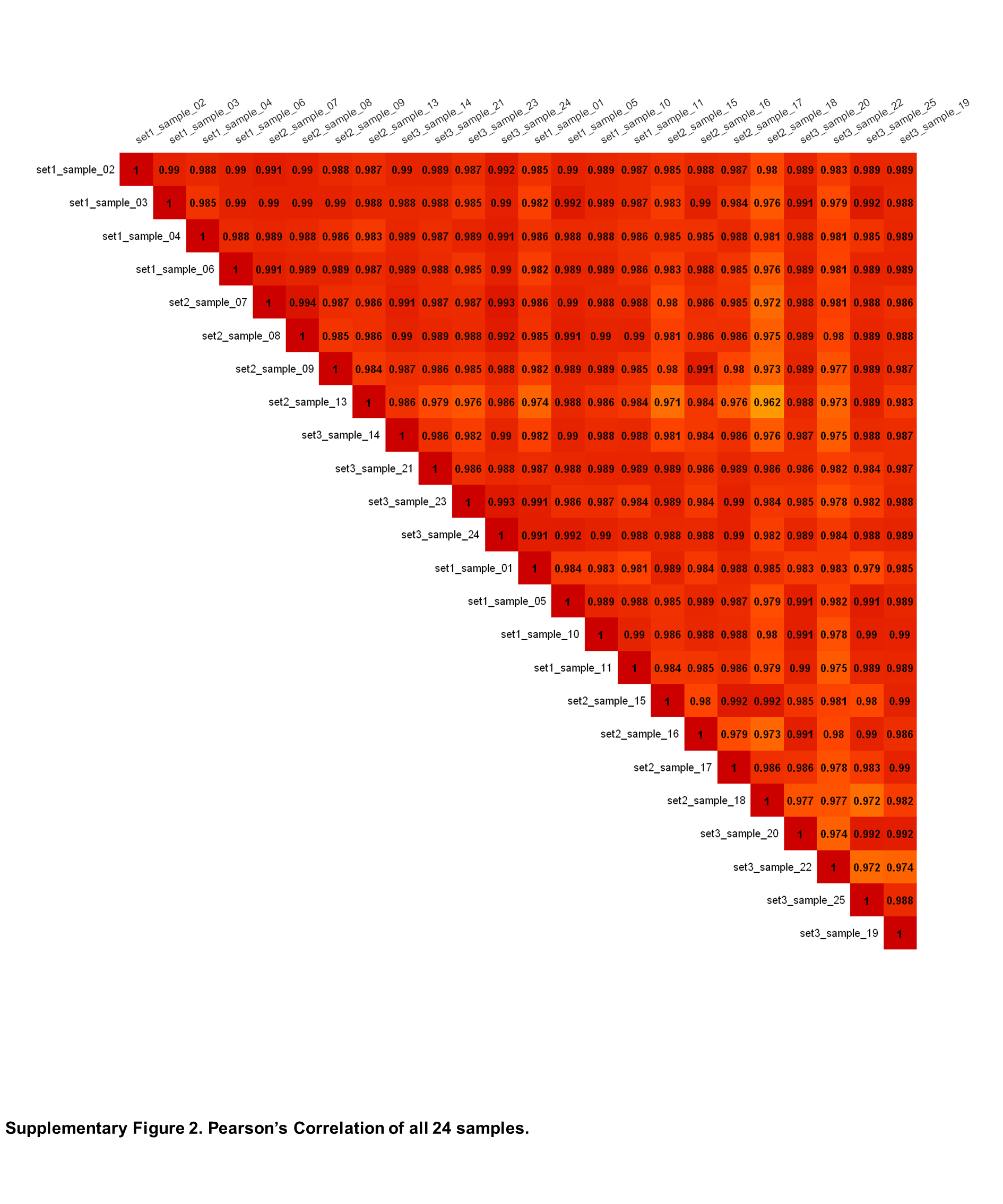

Supplement: Supplementary file 2 — Supporting information [file CTM2-14-e1693-s008.PNG]

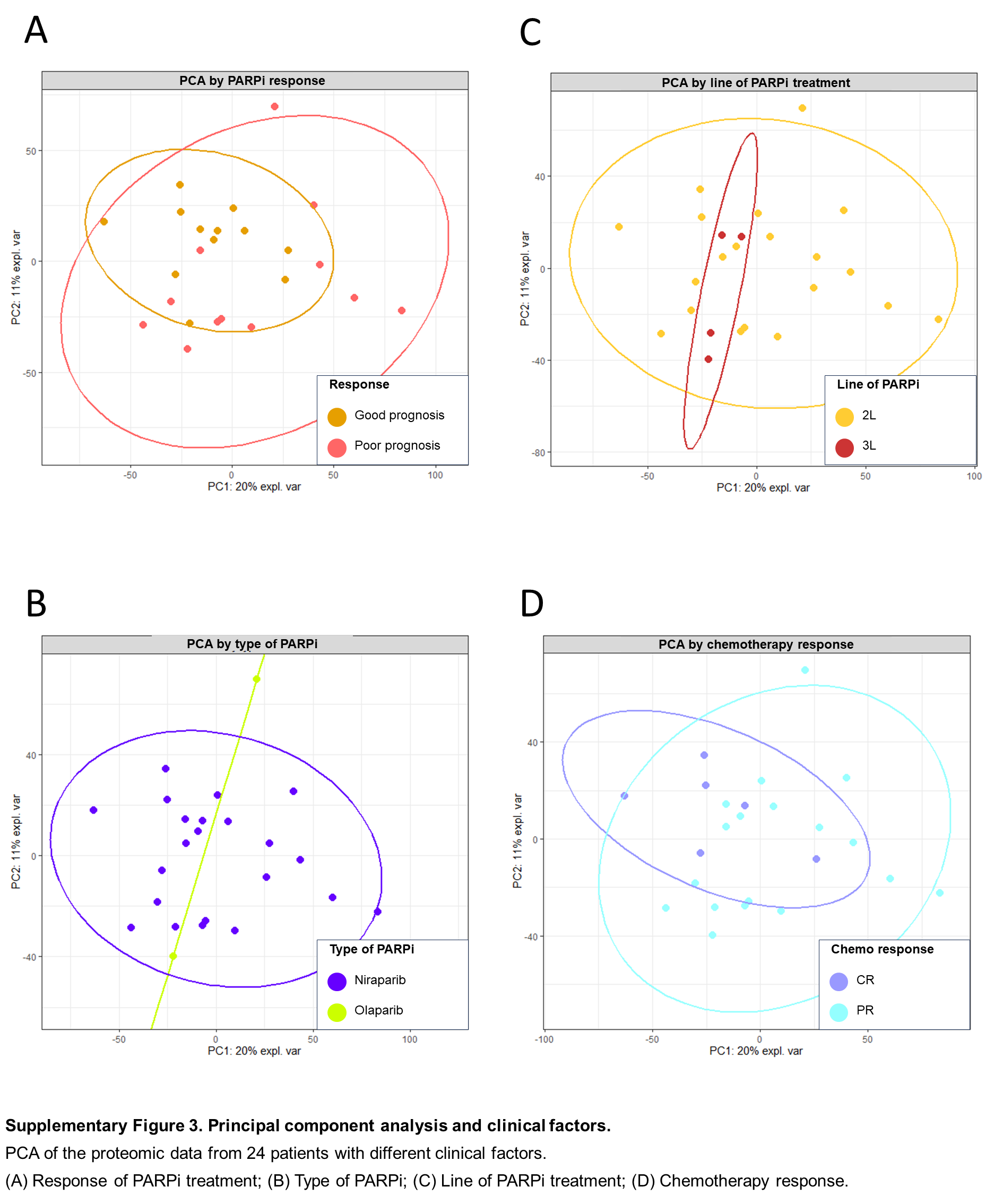

Supplement: Supplementary file 3 — Supporting information [file CTM2-14-e1693-s001.PNG]

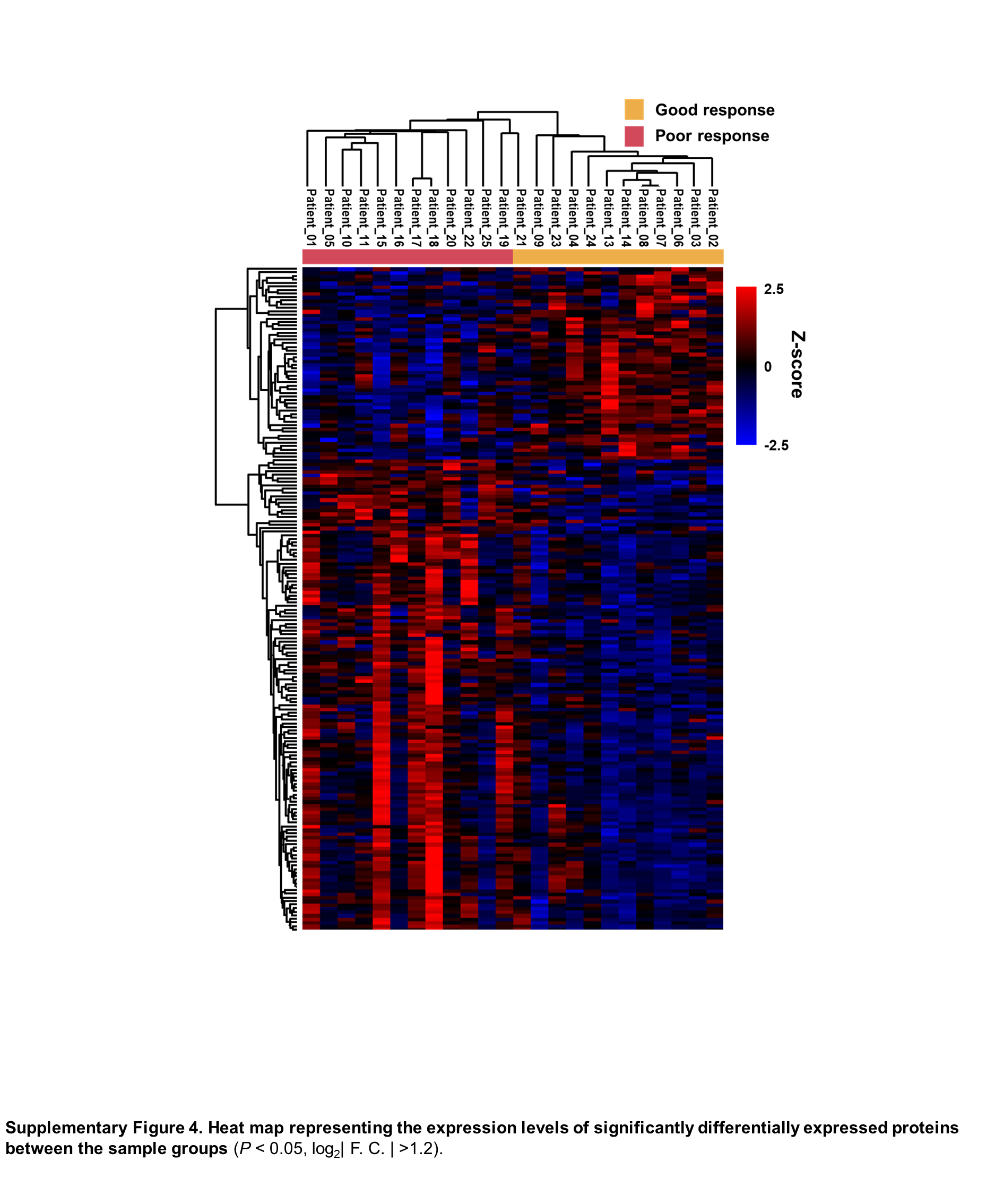

Supplement: Supplementary file 4 — Supporting information [file CTM2-14-e1693-s009.PNG]

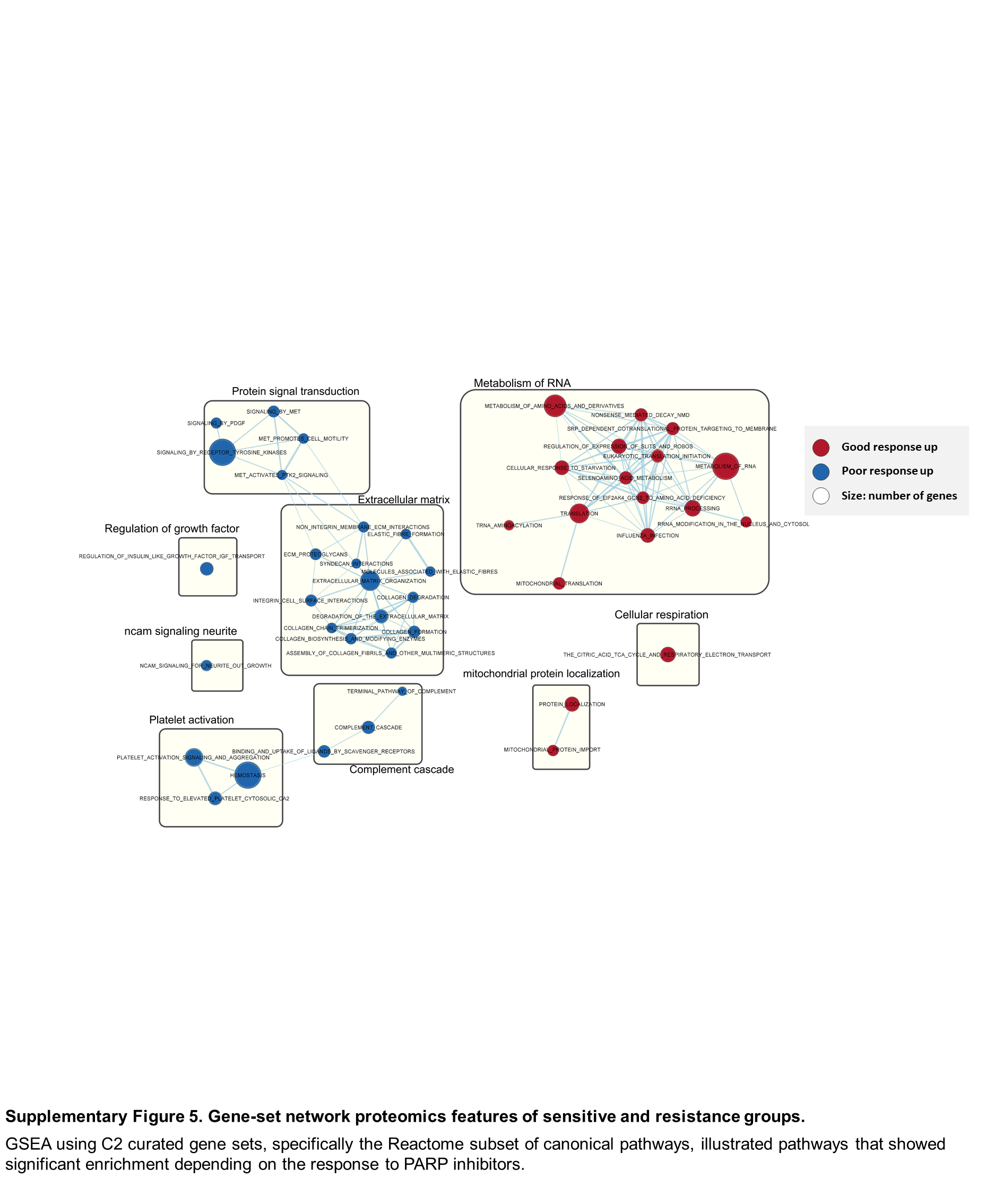

Supplement: Supplementary file 5 — Supporting information [file CTM2-14-e1693-s005.PNG]

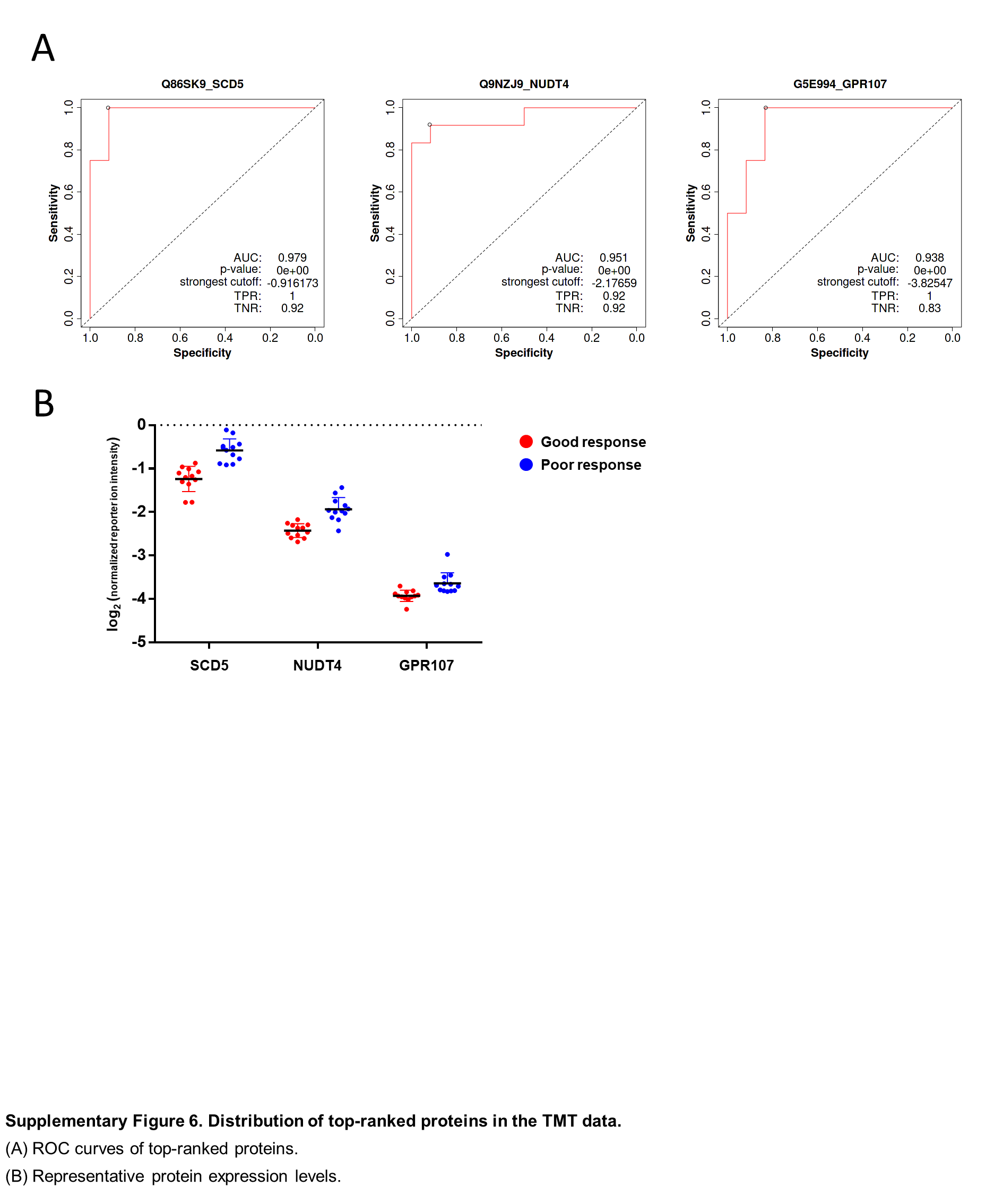

Supplement: Supplementary file 6 — Supporting information [file CTM2-14-e1693-s004.PNG]

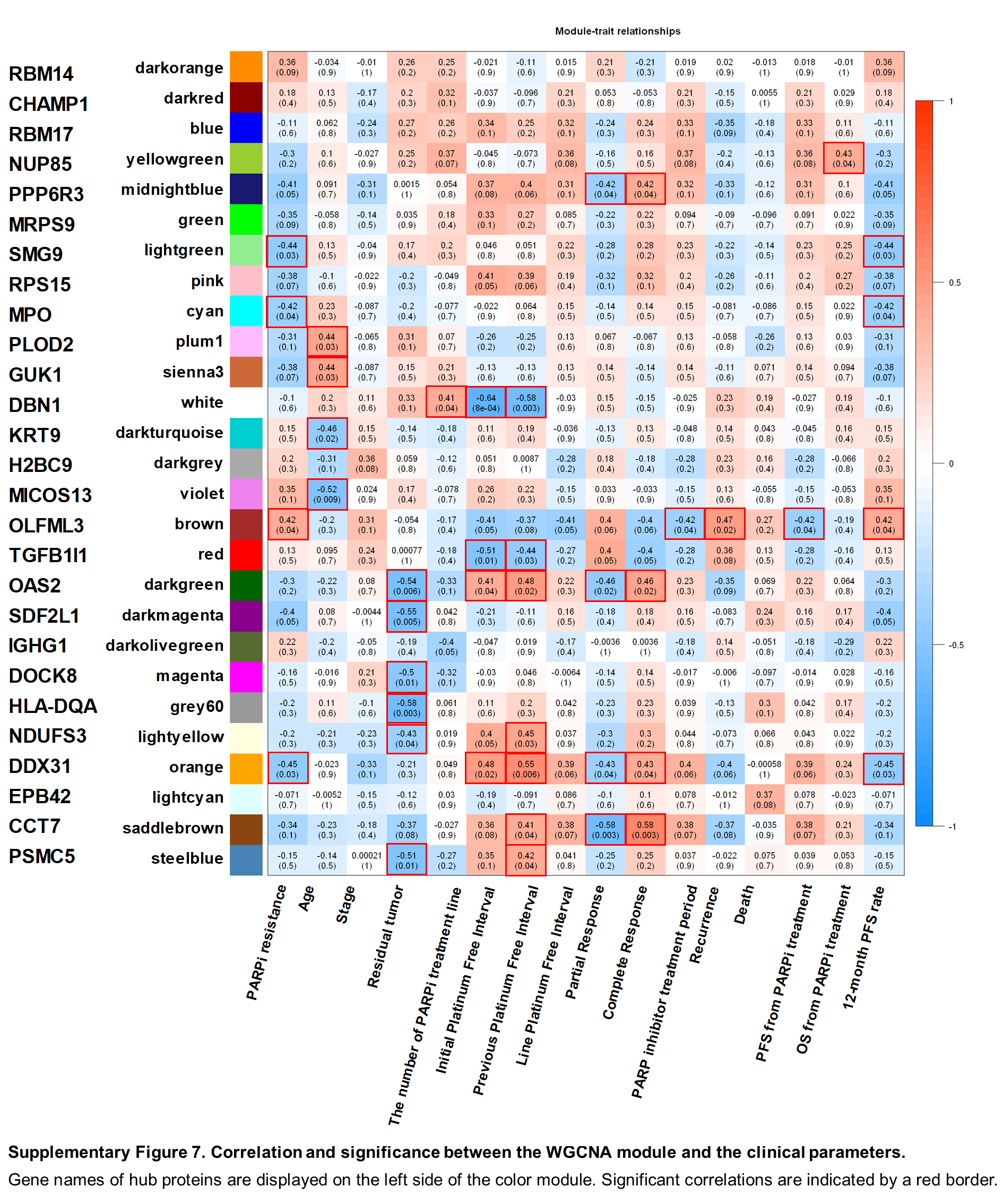

Supplement: Supplementary file 7 — Supporting information [file CTM2-14-e1693-s010.PNG]

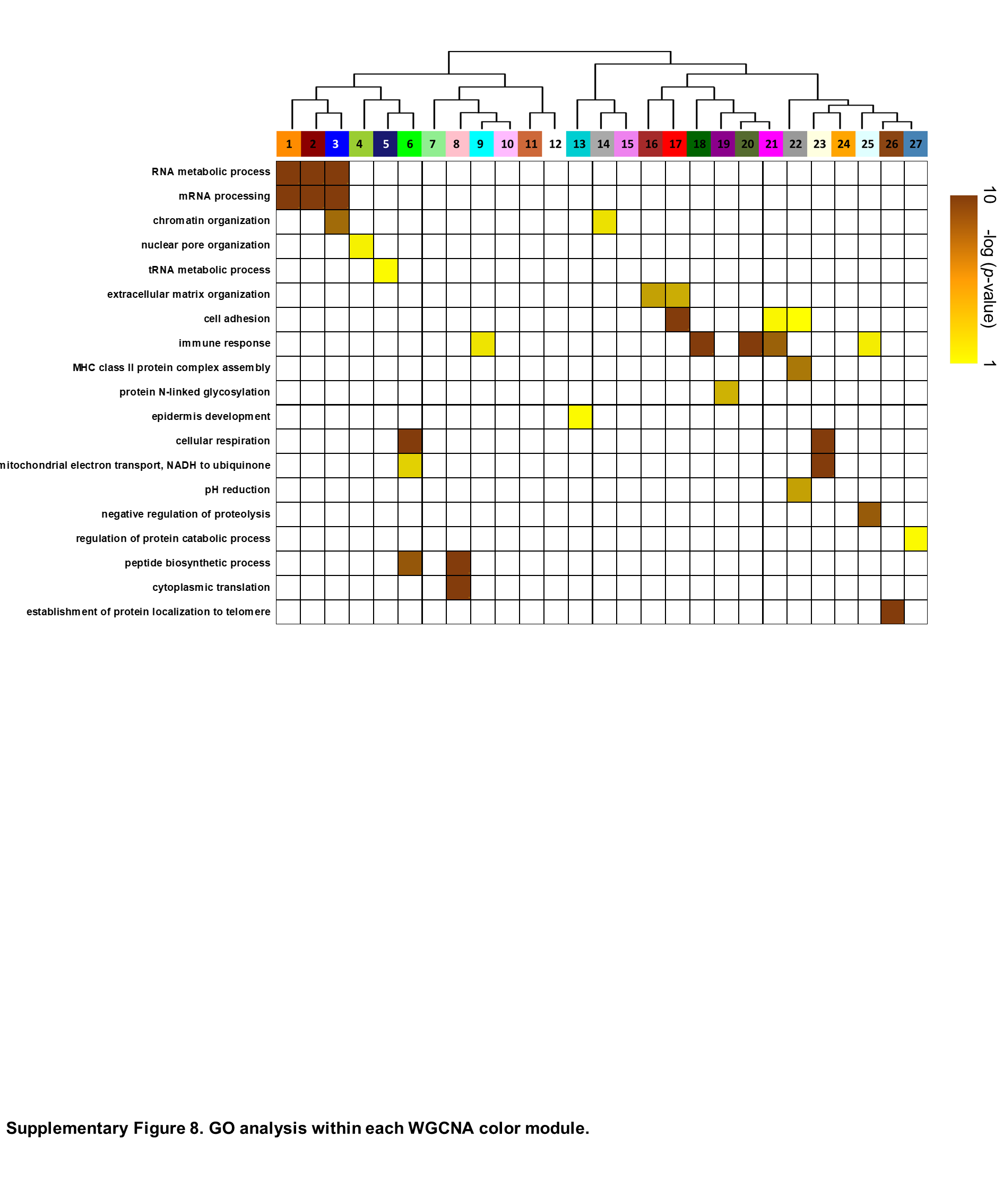

Supplement: Supplementary file 8 — Supporting information [file CTM2-14-e1693-s003.PNG]

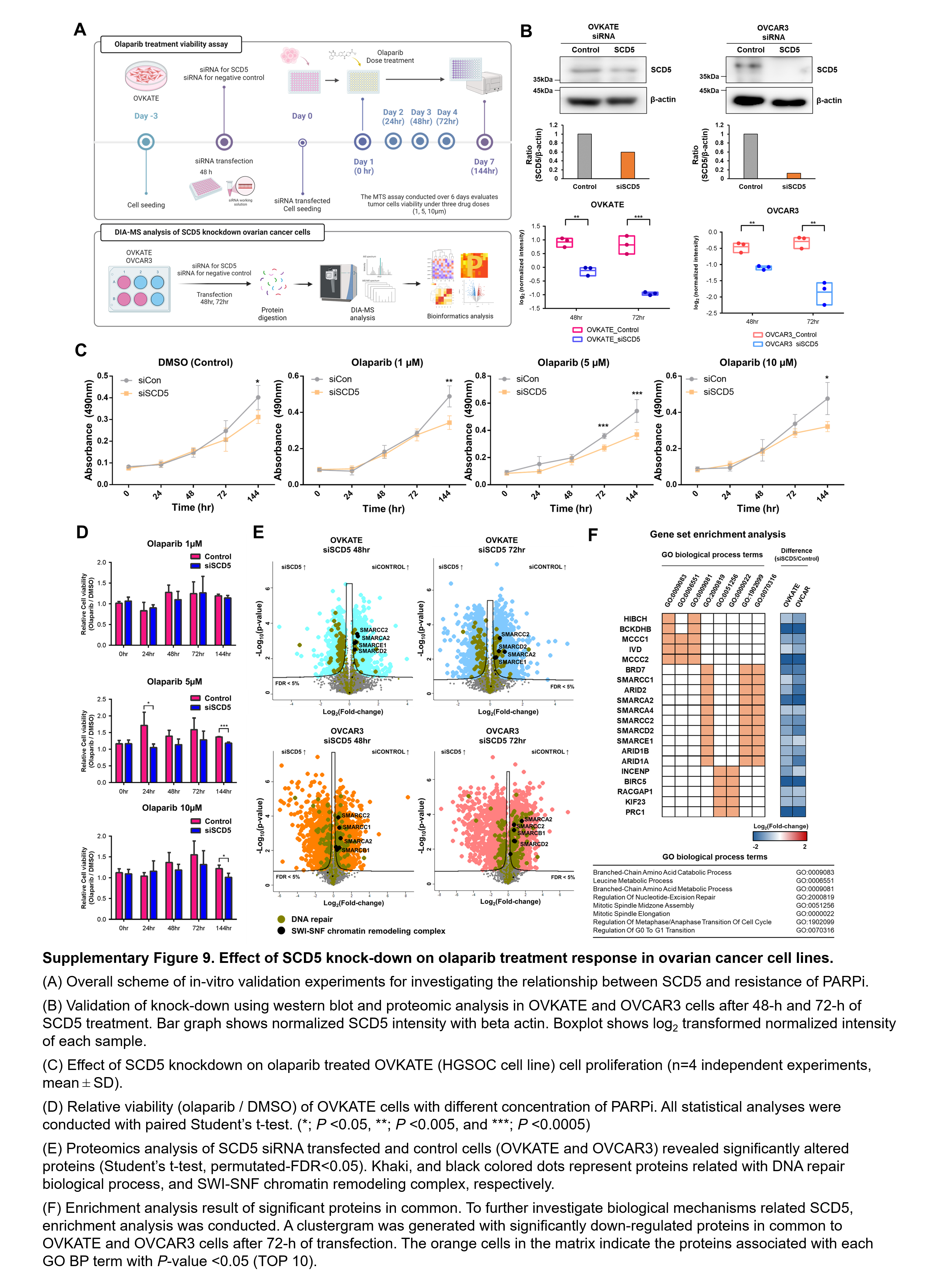

Supplement: Supplementary file 9 — Supporting information [file CTM2-14-e1693-s006.png]

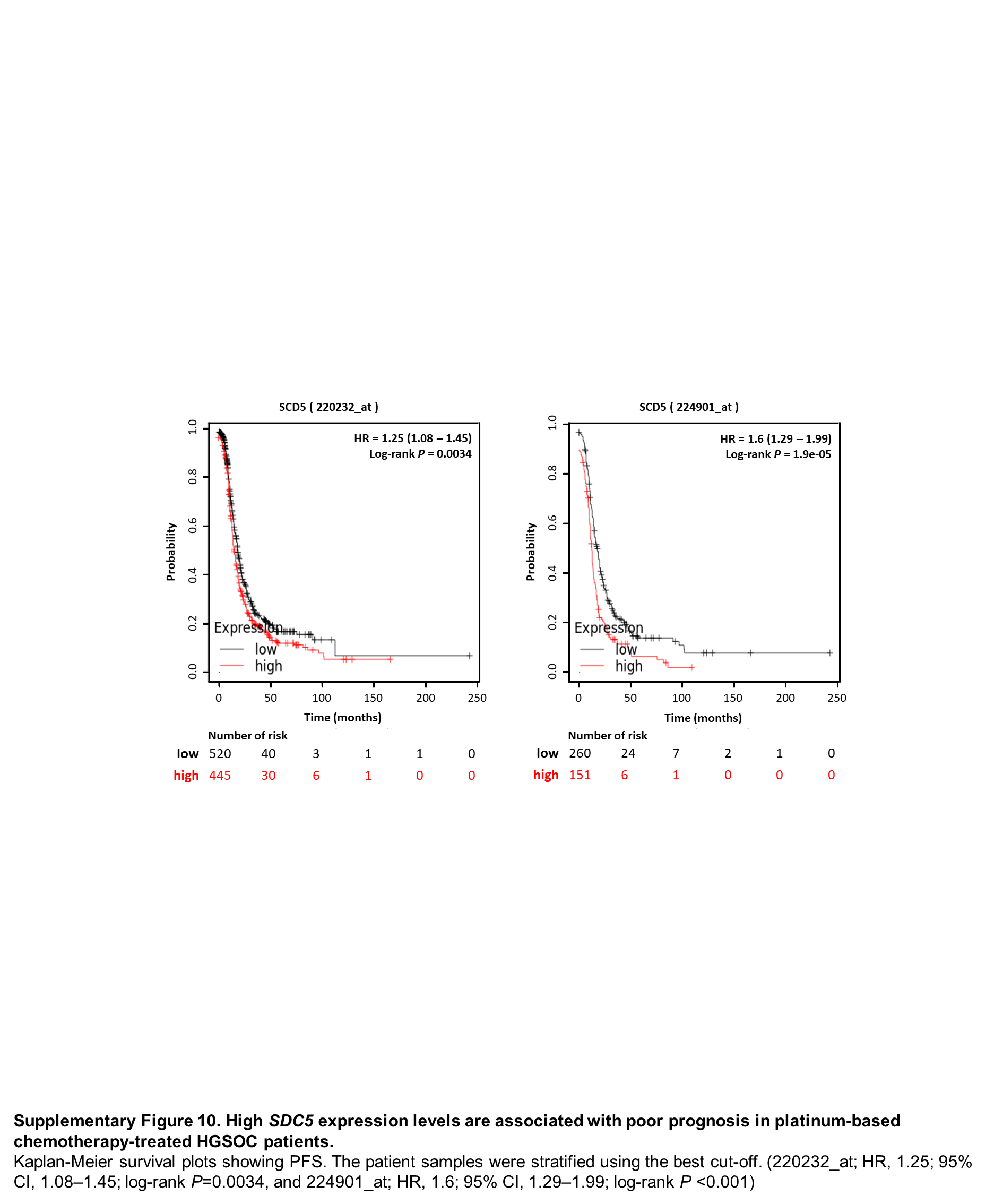

Supplement: Supplementary file 10 — Supporting information [file CTM2-14-e1693-s002.PNG]
