# Supplementary material for: Proteomic landscaping of high‐grade serous ovarian carcinoma identifies stearoyl‐CoA desaturase 5 as a potential predictive biomarker for poly(ADP‐ribose) polymerase inhibitor response
Source: Clin Transl Med. 2024 May 8;14(5):e1693. doi: 10.1002/ctm2.1693 (PMC11079157; doi:10.1002/ctm2.1693)
Supplement: Supplementary file 11 — Supporting information [file CTM2-14-e1693-s012.docx]

**Supplementary Information**

**Materials and Methods**

***Study population and data collection***

This study was approved by the Institutional Review Board of Seoul National University Hospital (No. H-2011-127-1173). We conducted the study in accordance with the principles of the Declaration of Helsinki and its amendments.

Considering the sociomedical environment of Korea (1), we established patients with *BRCA1/2* mutated, platinum-sensitive recurrent (PSR) HGSOC who received maintenance therapy with olaparib or niraparib inhibitors as the study population. We identified patients who met the following inclusion criteria: (1) aged ≥18 years at initial diagnosis; (2) histologically confirmed HGSOC; (3) received CRS and platinum-based chemotherapy as the primary treatment; (4) identified as having a deleterious/suspected deleterious *BRCA1/2* mutation at germline/somatic testing; (5) relapse ≥6 months after the last administration of platinum-based chemotherapy (i.e., PSR); (6) responded (complete response [CR] or partial response [PR]) to 4–9 cycles of second- or third-line platinum-based combination chemotherapy; (7) started maintenance therapy with either olaparib (capsules 400 mg bid orally or tablets 300 mg bid orally) or niraparib (capsules 200 mg qd orally for patients with baseline body weight <77 kg or platelet count <150,000/μL; otherwise, capsules 300 mg qd orally) between January 2018 and March 2021; and (8) whose FFPE primary ovarian cancer tissues were stored at Department of Pathology for scientific purposes. Patients were excluded if they (1) had any malignancy other than ovarian cancer, (2) had received frontline PARPi maintenance therapy, (3) had received PARPi maintenance therapy for less than 12 months without progression but were lost to follow-up or discontinued due to unacceptable toxicity or refusal of the patient, or (4) had insufficient clinicopathologic data.

Through a review of medical records, we collected baseline characteristics and treatment-related and survival data. Reduction or interruption of the PARPi dose was allowed at the discretion of the physician and continued until disease progression. During and after PARPi maintenance therapy, the patients underwent surveillance with routine computed tomography (CT) scans every three months. Disease progression was evaluated using CT scans according to the Response Evaluation Criteria in Solid Tumors version 1.1 (2). Progression-free survival (PFS) and overall survival were defined as the time intervals from the initiation date of PARPi maintenance therapy to the date of disease progression and to the date of cancer-related death or last visit, respectively.

Clinicopathologic characteristics were compared between the two groups using Student’s t-test or Mann–Whitney *U* test for continuous variables and Pearson’s chi-square test or Fisher’s exact test for categorical variables. Statistical significance was set at *P* <0.05.

***Cell culture***

The ovarian cancer cell lines OVKATE and OVCAR3 were maintained at 37°C in a humidified atmosphere containing 5% CO2 in RPMI supplemented with 10% fetal bovine serum (FBS; Gibco, Rockford, IL, USA) and 1% Pen Strep (Gibco, Rockford, IL, USA).

***siRNA transfection***

For the knockdown experiment, siRNAs specific for human SCD5 and negative controls were purchased from Bioneer (Daejeon, Korea) or Genepharma (Shanghai, China). The sequences of sense and antisense oligonucleotides for CD44 siRNA were as follows: sense, 5′-GGA GAA AGC UUG ACG UCA CUG-3′; antisense, 5′-CAG UGA CGU CAA GCU UUC UCC -3′. The sequences of sense and antisense oligonucleotides for the negative control were as follows: sense, 5′-UUC UCC GAA CGU GUC ACG UTT-3′; antisense, 5′-ACG UGA CAC GUU CGG AGA ATT-3′. For the proteomics analysis, 3 × 10^6^ cells were plated in 6-well culture plates and transfected with 50 nM of negative control or SCD5 siRNA using Lipofectamine RNAiMAX (Invitrogen, Carlsbad, CA, USA) according to the manufacturer’s procedure. Transfected cells were analyzed after incubation with the siRNA complex for 48 and 72 hours.

***Western blotting***

Proteins were extracted from cells using RIPA buffer (Sigma-Aldrich, St. Louis, MO, USA) combined with a protease inhibitor cocktail (Roche, Basel, Switzerland). Lysates of proteins were subjected to SDS-PAGE and were transferred to a PVDF membrane (Millipore, Darmstadt, Germany). The membrane was blocked in 5% BSA (BD, San Jose, CA) in TBS-T (0.05% (v/v) tween-20 in tris-buffered saline) and incubated with primary antibodies for SCD5 (NBP2-94369; NOVUS Biologicals, Centennial, CO, USA) and β-actin (sc-47778; Santa Cruz Biotechnology, Santa Cruz, CA, USA). After washing in TBS-T, the membrane was incubated with horseradish peroxidase-conjugated secondary antibodies against rabbit for SCD5 or mouse for β-actin. After washing in TBS-T, protein expression was detected with a biomolecular imager (GE, Boston, MA) using the membranes and an ECL solution (AB Frontier, Seoul, Korea).

***Drug treatment and viability assay***

For the viability assay according to drug treatment, 3 × 10^4^ cells were seeded in 4-5 replicates onto 96-well culture plates (SPL, Seoul, Korea) for each condition. After a 24-h cultivation, the cells were transfected with negative control and SCD5 siRNA. After incubation for 48-hour, 5 x 10^3^ of the siRNA transfected cells were seeded onto new 96-well culture plates. The viability of cells was assessed using the CellTiter 96 AQueous One Solution Cell Proliferation Assay (Promega, Madison, WI, USA) after drug treatment for 24, 48, 72, and 144 hours using 1, 5, 10µm of olaparib (Selleckchem, Houston, TX, USA). After treatment with 20 μL of One Solution reagent containing MTS [3-(4,5-dimethylthiazol-2-yl)-5-(3-carboxymethoxyphenyl)-2-(4-sulfophenyl)-2H-tetrazolium], each cell sample was incubated with 150 μL of fresh medium for 2 h at 37 °C with 5% CO2. The absorbance was measured at a wavelength of 490 nm.

***FFPE tissue preparation***

We used anonymized chemotherapy-naïve archival FFPE primary HGSOC tissues. After retrieval, the FFPE tissue blocks were sliced into 10-µm thick sections. The corresponding slides stained with hematoxylin and eosin were reviewed by a pathologist (Cheol Lee), an expert in gynecologic oncology. Tumor portions containing at least 70% of the cellular areas were marked, whereas fibrotic and necrotic areas were avoided. The slides were deparaffinized with Neo-Clear™ (Sigma-Aldrich, 1098435000, USA) and rehydrated with graded ethanol and water. Referring to the marked part as a template, the tumors were collected using a scalpel blade and lysed with an SDS extraction buffer (4% SDS, 300 mM Tris-HCl, pH 7.4, and 1 mM TCEP). After sonication, the samples were boiled at 95 °C. Protein samples were precipitated using cold acetone. The pellets were resuspended in lysis buffer (5% SDS, 10 mM TCEP, and 50 mM CAA in 100 mM Tris, pH 8.5) and incubated at 95 °C for 15 min. The samples were then digested using S-Trap™ micro columns (Protifi, C-02-micro, NY, USA), according to the manufacturer’s instructions. Briefly, samples acidified with aqueous phosphoric acid were resuspended in strap buffer (100 mM triethylammonium bicarbonate in 90% methanol) and added to the strap columns. After centrifugation (3,000 rcf), the flow was discarded, and the columns were washed five times with strap buffer. Digestion was performed by adding a Trypsin/LysC Mixture (Promega, V5071, WI, USA) to the columns, which were incubated at 47 °C for 2 h. The samples were eluted using sequentially graded acetonitrile (ACN) in 0.1% formic acid. The eluted fractions were pooled and dried, and the peptide concentrations were quantified based on tryptophan fluorescence (3). Samples for DIA were desalted using homemade SDB-RPS stage tips, as previously explained (4).

***TMT labelling***

For relative quantification, the peptides were labelled in three groups of TMT 10-plex (Thermo Scientific, 90110, MA, USA) experiments as described previously (5). The pooled peptides were labelled with 130C and 131 as internal references for each TMT experiment. The well-responding patient-derived peptides were labelled 126, 127N, 127C, and 128N. Peptides derived from poorly responding patients were labelled 128C, 129N, 129C, and 130N. The peptides used were 7 µg per sample and pooled peptides were prepared at 8 µg for each channel. Labelled peptides were dried in a speed-vac and desalted using a C18 OASIS HLB cartridge (30 mg, Waters, WAT094225, MA, USA) before HPLC fractionation.

***Offline high-pH reversed-peptide fractionation***

The TMT-labelled tryptic peptides were fractionated offline using the reversed-phase high-pH strategy described previously (5). Desalted peptides were subjected to Agilent 1290 bioinert HPLC (Agilent, Santa Clara, CA, USA) equipped with an analytical column (4.6 × 250 mm, 5 μm). For peptide separation, buffer A comprised 15 mM ammonium hydroxide and buffer B comprised 15 mM ammonium hydroxide in 90% ACN. The peptides were fractionated using a 5–35% ACN gradient at a flow rate of 0.2 mL/min. A total of 96 fractions were concatenated into 24 fractions and evaporated under vacuum.

***LC-MS/MS analysis***

All liquid chromatography (LC) with tandem MS (MS/MS) analyses were conducted using an Ultimate 3000 UHPLC system (Dionex, Sunnyvale, CA, USA) coupled to an Orbitrap Exploris (OE) 480 mass spectrometer (Thermo Scientific, Hamburg, Germany), as previously described with some modifications (6). Peptide samples were separated in a two-column system with a trap column (300 µm I.D × 5 mm length) and an analytical column (EASY-Spray C18, 75 µm I.D. × 50 cm length) with 180 min gradients from 8–30% acetonitrile at 300 nL/min. The column temperature was maintained at 60 °C using a column heater.

For TMT data acquisition, the fractionated samples were resuspended in loading buffer (2% ACN and 0.1% FA) and loaded onto a column. A survey scan (m/z 350–1650) was performed at a resolution of 120,000 and an m/z of 200. The top-20 method was used to select precursor ions with an isolation window of 0.7 m/z. The MS/MS spectrum was acquired at an HCD-normalized collision energy of 32 and a resolution of 35,000 at m/z 200. The maximum ion injection times for the full and MS/MS scans were 20 ms and 100 ms, respectively.

For the DIA data, peptides with iRT peptides (Biognosys, Schlieren, Switzerland) were analyzed. A full scan was acquired between m/z 350 and 1650 at a resolution of 120,000. The AGC target was 3e6 and the maximum injection time was set to 47 ms. A total of 41 segments were acquired at a resolution of 30,000 pixels. The normalized collision energies were stepped at 25.5, 27, and 30. The AGC target was 3 × 10^6^, and the maximum injection time was set to 55 ms. For library generation, data-dependent acquisition (DDA) was performed on the same samples. The top 15 methods used a scan range of 350–1650 m/z with a full mass resolution of 60,000. The AGC target was 3e6 or the maximum injection time was 20 ms. The dependent MS2 scan was performed at a resolution of 15,000 and an isolation window of 1.6. The AGC target was 2 × 10^6^, and the maximum injection time was 25 ms.

***Data processing***

For the database search for the TMT 10-plex experiment, raw MS files were processed using Proteome Discoverer ver 2.4 with the SEQUEST-HT algorithm against the Human UniProt protein sequence database (June 2021, 101,014 entries). The search parameters included full enzyme digestion using trypsin with up to two missed cleavages, 20-ppm peptide precursor mass tolerance, and fragment ion mass tolerance of 0.02 Da. Variable modifications of 15.995 Da for methionine oxidation and 42.011 Da for protein N-terminal acetylation, fixed modifications of 57.021 Da for carbamidomethylation of cysteine residues, 229.153 Da for TMT 10-plex-labelled lysine, and any N-terminus were selected. The co-isolation threshold for peptide quantification was set to 50%. The false discovery rates (FDRs) of the peptide spectral matches and proteins were set to <1%.

For DIA, data were analyzed with Spectronaut 17 (Biognosys) using the default setting (7). For library generation, DDA and DIA data were analyzed using the following parameters: full enzyme digestion was set to trypsin/P with up to two missed cleavages. The search criteria included carbamidomethylation of cysteine as a fixed modification, and oxidation of methionine and acetylation (protein N-terminus) as variable modifications. The raw files were searched against the same sequence database as the TMT database search and the Biognosys iRT peptide FASTA file. To process the DIA data, the Q-values of the peptides and proteins were set to <0.01.

***Quantification and statistical analysis***

Pre-processing and statistical analysis of proteomic data were performed using Perseus software (8). Initially, proteins identified as reverse hits and contaminants were removed. Reverse proteins, proteins identified only by site, and potential contaminants were excluded from the analysis. Protein expression levels were estimated by determining the logarithmic (log2(x)) intensities of the TMT-reporter ions. Valid values were filtered using proteins with quantified values in all TMT sets. After quantile normalization, the batch effect among the three TMT 10-plex sets was eliminated using the COMBAT algorithm (9). To identify differentially expressed proteins (DEPs), we applied Student’s t-test with a nominal p-value and a 1.2-fold cutoff (*P* <0.05, |fold-change| >1.2). DEP abundances were subjected to z-normalization, followed by hierarchical clustering using Pearson's correlation. Principal component analysis (PCA) was performed using the R statistical environment (version 4.1.3) with mixOmics package (10).

***Bioinformatics analysis***

For enrichment analysis using R-based EnrichR, a list of DEPs and the differences in each protein were used. The libraries of EnrichR gene sets referenced in the analysis were Gene Ontology (GO) terms for biological processes (BPs) (2021) and pathways from the Reactome (2016) database (11, 12). Statistical tests were performed using Fisher’s exact test based on multiple-testing correction. For the gene set enrichment analysis (GSEA), significantly expressed proteins and differences in each protein were used. The enriched terms from BP (C5 in MsigDB GO gene sets) and Reactome (C2 in MsigDB of the canonical pathway) were obtained using the EnrichmentMap plug-in of Cytoscape (version 3.9.1) (13). For this analysis, protein sets were extracted from the data using significant cutoffs, and protein expression values were ranked by fold change. The results were visualized with a Q-value <0.01. Kaplan–Meier survival analysis for PFS with chip mRNA data was performed using a Kaplan–Meier plotter to exclude outlier array quality control (14).

***Feature selection***

Feature selection was performed using the R Caret package. Herein, three machine learning (ML) classification algorithms were used to assess variable importance: Random forest, support vector machine (SVM), and extreme gradient boosting (XGBoost). Leave-one-out cross-validation was used to determine the importance of each protein and to reduce the error rate. In the random forest analysis, 3300 trees were built, which was repeated 108 times. A linear kernel implementation was used for the binary classification of the SVM. The XGB algorithm with a learning rate of 0.3 was repeated 50 times.

***Weighted gene co-expression network analysis***

Weighted gene co-expression network analysis (WGCNA) (version 1.71) was performed on all 24 samples using the R code (15). Before analysis, the samples were clustered using the hclust of the WGCNA package to identify significant outliers in the samples based on the average. To define the network modules, protein network construction was performed with a soft-thresholding power β of 12. Module identification was conducted using parameters corresponding to a minModuleSize of 30, deepSplit of 4, cutHeight of 0.25, threshPercent of 50, and mergePercent of 25. Functional enrichment analysis was performed for each protein module. Protein–protein interaction analysis was conducted using the STRING protein-protein interaction (PPI) databases (version 11.5) (16, 17). The network of co-expressed proteins was visualized using Cytoscape (version 3.9.1) (18).

**REFERENCES**

1. Kim SI, Lee M, Kim HS, Chung HH, Kim JW, Park NH, et al. Germline and Somatic BRCA1/2 Gene Mutational Status and Clinical Outcomes in Epithelial Peritoneal, Ovarian, and Fallopian Tube Cancer: Over a Decade of Experience in a Single Institution in Korea. Cancer Res Treat. 2020;52(4):1229-41.

2. Eisenhauer EA, Therasse P, Bogaerts J, Schwartz LH, Sargent D, Ford R, et al. New response evaluation criteria in solid tumours: revised RECIST guideline (version 1.1). Eur J Cancer. 2009;45(2):228-47.

3. Wiśniewski JR, Gaugaz FZ. Fast and sensitive total protein and Peptide assays for proteomic analysis. Anal Chem. 2015;87(8):4110-6.

4. Kulak NA, Pichler G, Paron I, Nagaraj N, Mann M. Minimal, encapsulated proteomic-sample processing applied to copy-number estimation in eukaryotic cells. Nature methods. 2014;11(3):319-24.

5. Kim H, Woo J, Dan K, Lee KM, Jin MS, Park IA, et al. Quantitative Proteomics Reveals Knockdown of CD44 Promotes Proliferation and Migration in Claudin-Low MDA-MB-231 and Hs 578T Breast Cancer Cell Lines. Journal of proteome research. 2021;20(7):3720-33.

6. Kim JE, Han D, Jeong JS, Moon JJ, Moon HK, Lee S, et al. Multisample Mass Spectrometry-Based Approach for Discovering Injury Markers in Chronic Kidney Disease. Mol Cell Proteomics. 2021;20:100037.

7. Bruderer R, Bernhardt OM, Gandhi T, Miladinović SM, Cheng LY, Messner S, et al. Extending the limits of quantitative proteome profiling with data-independent acquisition and application to acetaminophen-treated three-dimensional liver microtissues. Mol Cell Proteomics. 2015;14(5):1400-10.

8. Tyanova S, Temu T, Sinitcyn P, Carlson A, Hein MY, Geiger T, et al. The Perseus computational platform for comprehensive analysis of (prote)omics data. Nature methods. 2016;13(9):731-40.

9. Kim C, Wang XD, Yu Y. PARP1 inhibitors trigger innate immunity via PARP1 trapping-induced DNA damage response. Elife. 2020;9.

10. Rohart F, Gautier B, Singh A, KA LC. mixOmics: An R package for 'omics feature selection and multiple data integration. PLoS Comput Biol. 2017;13(11):e1005752.

11. Kuleshov MV, Jones MR, Rouillard AD, Fernandez NF, Duan Q, Wang Z, et al. Enrichr: a comprehensive gene set enrichment analysis web server 2016 update. Nucleic acids research. 2016;44(W1):W90-7.

12. Chen EY, Tan CM, Kou Y, Duan Q, Wang Z, Meirelles GV, et al. Enrichr: interactive and collaborative HTML5 gene list enrichment analysis tool. BMC bioinformatics. 2013;14:128.

13. Reimand J, Isserlin R, Voisin V, Kucera M, Tannus-Lopes C, Rostamianfar A, et al. Pathway enrichment analysis and visualization of omics data using g:Profiler, GSEA, Cytoscape and EnrichmentMap. Nature protocols. 2019;14(2):482-517.

14. Győrffy B. Discovery and ranking of the most robust prognostic biomarkers in serous ovarian cancer. GeroScience. 2023.

15. Langfelder P, Horvath S. WGCNA: an R package for weighted correlation network analysis. BMC bioinformatics. 2008;9:559.

16. von Mering C, Huynen M, Jaeggi D, Schmidt S, Bork P, Snel B. STRING: a database of predicted functional associations between proteins. Nucleic acids research. 2003;31(1):258-61.

17. Szklarczyk D, Gable AL, Lyon D, Junge A, Wyder S, Huerta-Cepas J, et al. STRING v11: protein-protein association networks with increased coverage, supporting functional discovery in genome-wide experimental datasets. Nucleic acids research. 2019;47(D1):D607-d13.

18. Shannon P, Markiel A, Ozier O, Baliga NS, Wang JT, Ramage D, et al. Cytoscape: a software environment for integrated models of biomolecular interaction networks. Genome research. 2003;13(11):2498-504.

**Supplementary Figure 1. Survival analysis of the discovery cohort.**

(Upper) Progression-free survival; (Lower) Overall survival.

(A, C) All patients; (B, D) Comparisons between the good and poor response groups.

**Supplementary Figure 2. Pearson’s Correlation of all 24 samples.**

**Supplementary Figure 3. Principal component analysis and clinical factors.**

PCA of the proteomic data from 24 patients with different clinical factors.

(A) Response of PARPi treatment; (B) Type of PARPi; (C) Line of PARPi treatment; (D) Chemotherapy response.

**Supplementary Figure 4. Heat map representing the expression levels of significantly differentially expressed proteins between the sample groups** (*P* <0.05, log_2_| F. C. | >1.2).

**Supplementary Figure 5. Gene-set network proteomics features of sensitive and resistance groups.**

GSEA using C2 curated gene sets, specifically the Reactome subset of canonical pathways, illustrated pathways that showed significant enrichment depending on the response to PARP inhibitors.

**Supplementary Figure 6. Distribution of top-ranked proteins in the TMT data.**

(A) ROC curves of top-ranked proteins.

(B) Representative protein expression levels.

**Supplementary Figure 7. Correlation and significance between the WGCNA module and the clinical parameters.**

Gene names of hub proteins are displayed on the left side of the color module. Significant correlations are indicated by a red border.

**Supplementary Figure 8. GO analysis within each WGCNA color module.**

**Supplementary Figure 9. Effect of SCD5 knock-down on olaparib treatment response in ovarian cancer cell lines.**

(A) Overall scheme of in-vitro validation experiments for investigating the relationship between SCD5 and resistance of PARPi.

(B) Validation of knock-down using western blot and proteomic analysis in OVKATE and OVCAR3 cells after 48-h and 72-h of SCD5 treatment. Bar graph shows normalized SCD5 intensity with beta actin. Boxplot shows log_2_ transformed normalized intensity of each sample.

(C) Effect of SCD5 knockdown on olaparib treated OVKATE (HGSOC cell line) cell proliferation (n=4 independent experiments, mean ± SD).

(D) Relative viability (olaparib / DMSO) of OVKATE cells with different concentration of PARPi. All statistical analyses were conducted with paired Student’s t-test. (*; *P* <0.05, **; *P* <0.005, and ***; *P* <0.0005)

(E) Proteomics analysis of SCD5 siRNA transfected and control cells (OVKATE and OVCAR3) revealed significantly altered proteins (Student’s t-test, permutated-FDR<0.05). Khaki, and black colored dots represent proteins related with DNA repair biological process, and SWI-SNF chromatin remodeling complex, respectively.

(F) Enrichment analysis result of significant proteins in common. To further investigate biological mechanisms related SCD5, enrichment analysis was conducted. A clustergram was generated with significantly down-regulated proteins in common to OVKATE and OVCAR3 cells after 72-h of transfection. The orange cells in the matrix indicate the proteins associated with each GO BP term with *P*-value <0.05 (TOP 10).

**Supplementary Figure 10. High *SDC5* expression levels are associated with poor prognosis in platinum-based chemotherapy-treated HGSOC patients.**

Kaplan-Meier survival plots showing PFS. The patient samples were stratified using the best cut-off (220232_at; HR, 1.25; 95% CI, 1.08–1.45; log-rank *P*=0.0034, and 224901_at; HR, 1.6; 95% CI, 1.29–1.99; log-rank *P* <0.001).
